# Clinical attendance rate at a tertiary adult audiological service in South Africa

**DOI:** 10.4102/sajcd.v70i1.967

**Published:** 2023-11-14

**Authors:** Mubina Khan, Katijah Khoza-Shangase, Amanda B. Thusi, Ruhee Hoosain, Sadna Balton

**Affiliations:** 1 Department of Speech Therapy and Audiology, Chris Hani Baragwanath Hospital Speech Therapy and Audiology, Johannesburg, South Africa; 2 Department of Audiology, Faculty of Humanities, University of the Witwatersrand, Johannesburg, South Africa

**Keywords:** attendance rate, diagnostic audiology, clinic non-attendance, early intervention, South Africa

## Abstract

**Background:**

Clinical non-attendance to audiological appointments may negatively affect early diagnosis and intervention as well as treatment outcomes for adults with hearing impairments.

**Objectives:**

This study aimed to explore the attendance rate and factors influencing attendance and non-attendance at an adult audiology diagnostic clinic at a tertiary hospital in Gauteng, South Africa.

**Method:**

A mixed-methods research design, utilising structured questionnaires and a retrospective record review was adopted. A total of 31 adult patients at a diagnostic audiology clinic were interviewed.

**Results:**

Findings revealed an attendance rate of 47.62%, with 52.38% rate failure to return for follow-up appointments. Key reasons for attendance included understanding the need for appointments (57%), staff attitudes (42%) and appointment reminders (17%), and those for non-attendance included multiple appointments (33%), work commitments (28%), transport (8%) and forgetting about the appointment (8%). Six reasons for non-attendance were prominent in the current study: having multiple appointments (33%), work commitments (28%), forgetting the appointment (8%), transport difficulties (8%), attitudes and/or perceptions of the healthcare system (4%) and sequelae of hearing impairment (8%).

**Conclusion:**

This study reinforces previous research findings while highlighting that health literacy and Batho Pele (people first) ethos by staff positively influence attendance.

**Contribution:**

Current findings contribute towards contextually relevant evidence on the attendance rate in this sector for ear and hearing care, as well as additional insights into factors influencing this within the South African context. This information is crucial for clinical services provision planning as well as for policy formulation around resource allocation in the public healthcare sector.

## Introduction

South Africa has a population of approximately 55.7 million, with 26% of this population residing in the Gauteng province (Statistics South Africa, 2022). The country faces a quadruple burden of disease, which is four times higher than high-income countries (HICs) and two times higher than low- to middle-income countries (LMICs) (Lalkhen & Mash, 2015). The South African healthcare system aims to prevent illness and disease by promoting a healthy status and lifestyle (Department of Health, 2018). Healthcare delivery systems consistently strive to improve access, equity, quality and sustainability of services (Department of Health, 2020). *Batho Pele* (people first) principles are declared a national government White Paper for Transforming Public Service Delivery that emphasises the need for placing ‘People First’ (Frost et al., 2017; Maphumulo & Bhengu, 2019). The right to accessible and affordable healthcare is further enforced by the Constitution of South Africa and the National Development Plan (Maphumulo & Bhengu, 2019; Stellenberg, 2015). These aspects are in line with improving service delivery to the South African public (Education and Training Unit, 2017). Despite all these programmes, Malakoane et al. (2020) claim that South Africa’s public health programme performance and outcomes remain consistently inferior comparatively while the burden of disease continues to multiply.

The public healthcare sector in South Africa faces many challenges such as the increased burden of disease, unequal distribution of resources and leadership and management crisis: over and above long waiting times, rushed appointments, old infrastructure and poor disease control and prevention practices leading to healthcare-associated infections (Dramowski et al., 2017; Maphumulo & Bhengu, 2019; Young, 2016). The audiology profession within this sector is faced with capacity versus demand challenges as well as linguistic and cultural diversity incongruence when it comes to the match of the audiology workforce to the South African population (Pillay et al., 2020; Khoza-Shangase & Mophosho, 2018, 2021). Although patients are gradually becoming more aware of their right to quality healthcare, their non-compliance with keeping appointments can often be detrimental to the assessment and intervention outcomes as well as the efficient and cost-effective functioning of the healthcare system (Abuosi & Atinga, 2013; Kanji & Khoza-Shangase, 2018). The impact of patients’ non-compliance to their healthcare appointments may result in severe effects on treatment outcomes, increases in the financial burden for the community and a loss of productivity of healthcare staff and a wastage of resources (Geiger, 2015). These concerns are apparent across disciplines within healthcare (Kanji & Krabbenhoft, 2018; Khoza-Shangase, 2022a; Stellenberg, 2015). The current study specifically considers the attendance rate for audiology services within the public healthcare system in South Africa, with an exploration of influencing factors.

It is anticipated that by 2050, nearly 2.5 billion people will have hearing loss (Chadha et al., 2021) and will require intensive audiological management. A comparison between HICs and LMICs indicates that sub-Saharan Africa has a significantly higher prevalence rate of hearing loss. Available reports indicate an estimated prevalence of 11.5% – 20.3% for adults (≥ 15 years) compared to 4.0% – 6.4% for adults ≥ 15 years in HICs (Louw et al., 2018). The prevalence of acquired hearing loss in South Africa is affected by the high burden of disease, which has been dubbed the quadruple burden that includes burdens related to maternal, newborn and child health; human immunodeficiency viruses (HIV) and acquired immunodeficiency syndrome (AIDS) and Tuberculosis (TB); non-communicable diseases; as well as violence and injury (Khoza-Shangase, 2021; Louw et al., 2018). Mulwafu et al. (2016) argue that hearing loss is severely under-reported in South Africa. This was highlighted in a study conducted in a single metropolitan area in South Africa, where only 4.57% of disabling hearing impairment was reported in a population-based survey (Ramma & Sebothoma, 2016). The potential of increased numbers of individuals with hearing loss because of the burden of disease could potentially mean that audiology may become a service of much higher demand, which could further negatively impact access to ear and hearing care in LMICs (Chadha et al., 2021).

Hearing loss may present at any time along the life course of an individual (Chadha et al., 2021). An early, life course approach to audiological intervention as a key strategy to address the impact of hearing loss in adults is important (Chadha et al., 2020). This forms part of a preventive audiology paradigm that Khoza-Shangase (2022b) argues for within the African context. Early access to audiological services within the public healthcare sector is imperative for effective and efficient intervention, leading to positive cost-effective outcomes (Maluleke, 2022). The initiation of ear care and hearing loss intervention is dependent on patient attendance at their audiology appointments.

Attendance is defined as the action or state of being present at a place or event (Lexico, 2021). Non-attendance is defined as the lack of attending frequently or missing scheduled appointments (Henderson, 2008). The use of the word ‘rate’ of attendance and non-attendance is prominent in this article. Rate can be defined as the frequency at which something occurs (Lexico, 2021). For this study, rate refers to the frequency of attendance and non-attendance.

A United States (US)-based study by Kheirkhah et al. (2016) explored the rate of non-attendance at 10 outpatient specialties over 12 years, including audiology, and found a mean non-attendance rate of 18.8%, with audiology having the lowest non-attendance rate of 12.6%. Similarly, a study done by Asvat (2011) found that 33% of patients did not attend their physiotherapy appointments at a tertiary hospital in South Africa. Kanji and Khoza-Shangase (2018) have argued that careful strategies to address attendance rate within the South African audiology context are needed. Non-attendance leads to increased waiting times, decreased productivity for healthcare professionals because of unused appointment slots, wastage of resources and a financial burden on the healthcare facility (Geiger, 2015). Evidence from such studies highlights that non-attendance rate to outpatient appointments is much higher in South Africa than in globally based studies although much of these focus on the paediatric population (Hugill, 2021; Kanji & Krabbenhoft, 2018; Khoza-Shangase, 2021; Peter et al., 2020; Van der Spuy & Pottas, 2008).

Limited evidence exists on the attendance rate of adult patients for audiology appointments and the reasons for attendance and non-attendance within the public healthcare sector in South Africa, hence the importance of the current study. Investigating the reasons for non-attendance to appointments could provide much-needed insight into the challenges faced by patients as well as the changes required to enhance audiological service delivery to this already vulnerable population. Furthermore, such evidence could help improve maximum utilisation of the well-documented limited resources within the South African public healthcare system. This study, therefore, aimed to explore the attendance rate and factors influencing attendance and non-attendance at an adult audiology diagnostic clinic at a tertiary hospital in Gauteng, South Africa, in pursuit of positive preventive audiology outcomes.

## Research methods and design

### Aim

This study aimed to explore the attendance rate and factors influencing attendance and non-attendance at an adult audiology diagnostic clinic at a tertiary hospital in Gauteng, South Africa.

### Objectives

The specific objectives of this study were:

- to establish the attendance rate at the initial and follow-up appointments at the adult audiology diagnostic clinic;
- to identify reasons for attendance and non-attendance.

### Research design

A mixed-methods research design that used qualitative and quantitative methods, utilising structured questionnaires as an interview-based data collection method and a retrospective record review, was adopted for this study. This approach was adopted as it allowed for the gathering of specific and standardised information from participants in a consistent and efficient manner while considering patients’ reading and writing abilities. The structured questionnaire allowed for the systematic collection of data, enhancing the reliability and validity of the research findings. The interview allowed for the exploration of the reasons for attendance and non-attendance at the clinic. Furthermore, a retrospective record review of the audiology departmental appointment schedules, patient records and statistics was conducted to determine the attendance rate at the adult audiology diagnostic clinic.

### Site

The study was conducted at a tertiary hospital in Gauteng, South Africa, whose Audiology and Speech Therapy department provides in- and outpatient services across the lifespan. This hospital is a public healthcare facility servicing individuals who cannot afford private healthcare and falls under the healthcare sector that provides access to healthcare to over 80% of the South African population. Evidence suggests that only 16% of South Africans belong to medical aid schemes, allowing them access to the private healthcare sector for their healthcare needs (Naidoo 2012).

### Sampling strategy and sample size

Simple random sampling was utilised for adult patients attending their initial appointments, and purposive sampling was used for the patients who did not attend follow-up appointments. A convenience sampling method was utilised for the retrospective record review (appointment schedules, patient records and statistics) to establish the diagnostic clinic’s attendance and return rate from May 2019 to March 2020 (Etikan et al., 2016). A total of 31 adult patients were recruited ensuring representation of the diverse sociodemographic population seen at the clinic. This sample size was determined by data saturation, where participant recruitment was stopped the minute data saturation was confirmed for a category, and this was guided by the responses to the open-ended questions as well as questions where the interviewer had the flexibility to ask additional probing questions or seek clarification on certain responses to gain a deeper understanding of participants’ perspectives. This was defined as when no new data were obtained from participants in response to any question under the various categories (Boddy, 2016; Guest et al., 2006; Morse, 2000).

### Materials and methods

A structured questionnaire that was used as an interview-based data collection tool and a retrospective record review form was used. The structured questionnaire was carefully developed to ensure that it effectively captured the research objectives and addressed the research questions. The questionnaire included a set of predetermined questions and response options. The questionnaire contained mainly closed-ended questions, with a few open-ended questions focusing on the suggestions that participants might have that would help improve their attendance and/or reduce their non-attendance. The questionnaire consisted of five sections: (1) personal details, (2) length of time since onset of symptoms, (3) knowledge about audiology, (4) severity of symptoms and (5) reasons for non-attendance/attendance. With regard to exploring reasons for attendance and non-attendance within the questionnaire, patients were allowed to provide more than one reason from the list of reasons provided. The response options were derived from a thorough review of the existing literature, expert opinions and research objectives, and the five sections were designed to cover the key variables, concepts or themes related to the research topic. The questionnaire was translated into isiZulu as well as it is the most commonly spoken language in the area where the research site is located. IsiZulu is the home language to more than 24% of South Africa’s population and more than 50% of the population understand it (Ethnologue, 2022). The questionnaire was adapted from Asvat (2011) who used the tool in the study conducted at a physiotherapy department in a tertiary hospital in South Africa where adherence to attending appointments in an outpatient section was investigated.

Prior to the main data collection phase, a pilot study was conducted to assess the clarity, comprehensibility and relevance of the questionnaire. The pilot testing involved a small group of individuals who were similar to the target participants. Feedback from the pilot study was used to refine and improve the questionnaire to enhance validity and reliability, with each interview session following a standardised structure to ensure consistency across participants. The interview process commenced with an introduction, followed by an explanation of the purpose of the study and the confidentiality of participants’ responses. The structured questionnaire was then administered systematically, with the interviewer asking questions and recording participants’ responses. While using a structured questionnaire, the interviewer had the flexibility to ask additional probing questions or seek clarification on certain responses to gain a deeper understanding of participants’ perspectives. However, care was taken to maintain consistency across interviews and avoid excessive deviations from the structured questionnaire. To further enhance validity, member checking and peer debriefing were conducted.

### Data analysis

During data preparation, the data collected through the structured questionnaire were transcribed, coded and anonymised to ensure confidentiality and anonymity. The quantitative data collected through structured questionnaires were analysed using statistical software (e.g., Excel). Descriptive statistics, such as frequencies and percentages, were used to summarise the responses, with these presented in graphic and tabular forms (Cooksey, 2020; Kaur et al., 2018). Open-ended questions, and questions where the interviewer had the flexibility to ask additional probing questions or seek clarification on certain responses to gain a deeper understanding of participants’ perspectives, were analysed qualitatively via thematic analysis through a deductive approach where patterns, themes or emerging concepts were identified (Braun & Clarke, 2006).

### Ethical considerations

Prior to the study being conducted, ethical clearance was obtained from the University of the Witwatersrand’s Human Research Ethics Committee (Medical) (No. M1810644), and all relevant permissions were secured from the Head of the Department of Speech Therapy and Audiology and from the hospital’s research committee. The researchers adhered to all ethical considerations from the Declaration of Helsinki (1975 and revised in 2008).

## Results and discussion

Table 1 depicts profile descriptions for participants who either attended or did not attend follow-up appointments.

**TABLE 1:** Participant description.

Participant description	Attended (*n* = 20)		Non-attendance (*n* = 11)	
	*n*	%	*n*	%
**Age**				
Average age in years	45.5	-	53.6	-
**Gender**				
Male	4	20	3	27
Female	16	80	8	73
**Medical diagnoses**				
Acute	3	15	1	9
Chronic	15	75	9	82
None	2	10	1	9
**Length of hearing loss before audiological help was sought**				
Average amount of years	10.4	-	12.6	-
**Referral source**				
Ear, nose and throat doctor	9	45	5	5
Haematology doctor	2	10	0	0
Self	2	10	0	0
Local clinic	4	20	2	18
Unknown	3	3	4	36

Table 1 indicates that the average age of those who attended was younger (45.5 years), were more females, had more chronic medical diagnosis and were most likely referred from the Ear, Nose and Throat (ENT) specialist clinic, while the average age for those in the non-attendance group was older (53.6 years), also had more females than males, comprised more chronic medical diagnosis and were also from the ENT clinic. Besides the age difference between the two groups, a nuanced qualitative analysis of these factors seems to indicate that factors such as gender, medical diagnosis and referral source do not seem to influence attendance and non-attendance for this clinic. These findings are contrary to generally documented evidence showing that age, gender and medical diagnosis can influence clinic attendance and non-attendance (McLean et al., 2014; Thompson et al., 2016). For example, older adults have been documented to have higher attendance rate, because they have an increased prevalence of chronic health conditions and the need for regular monitoring or treatment.

Similarly, as far as gender is concerned, evidence has shown that women generally have higher rates of healthcare utilisation, including clinic attendance, because of factors such as reproductive health needs, pregnancy-related care and routine screenings (e.g., Pap smears, mammograms) (Staley et al., 2021). Furthermore, as far as medical diagnosis is concerned, specific medical diagnosis or health condition has been shown to significantly affect clinic attendance. Individuals with chronic conditions requiring ongoing medical management, such as diabetes, hypertension, cancer, TB or HIV and AIDS, often have regular clinic visits scheduled for treatment, monitoring or medication adjustments. In these cases, the diagnosis itself acts as a motivator for clinic attendance (McLean et al., 2014). Conversely, individuals with less severe or acute conditions might be more prone to non-attendance, particularly if they perceive their symptoms as mild or if they have concerns about the cost or inconvenience of seeking care (McLean et al., 2014).

In this study, current authors argue that it is important to be constantly aware that while age, gender and medical diagnosis have been shown previously to influence clinic attendance and non-attendance, these factors interact with numerous other individual, social and environmental factors, particularly in a socioeconomically unequal society like South Africa. Financial constraints, transportation accessibility, health literacy, cultural beliefs and the availability of health care services in a given area are just a few additional factors that may have impacted the current profile.

### Attendance and reasons for attendance

Adult diagnostic assessments at this institution run daily with an average of 12 patients being scheduled per day. An analysis of patient attendances from May 2019 to March 2020 revealed an attendance rate of 84% for first-time initial consultations.

This high attendance rate for initial appointments as opposed to decrease follow-up attendance rate is not surprising. Initial appointments have been reported to have higher attendance rates when compared to follow-up appointments. As noted by McLean et al. (2014) and Wolff et al. (2019), numerous reasons have been proffered for this evidence, including factors such as: (1) urgency and symptom severity, where it is reported that patients will prioritise seeking healthcare when experiencing acute or severe symptoms, thus the urgency of the situation motivating them to attend the initial appointment to address their immediate health concerns; (2) diagnostic investigation, where initial appointments are often for diagnostic investigations or assessments to determine the cause of the symptoms or to establish a baseline for ongoing care, as it is often the case with ENT referred patients, thus patients may feel more compelled to attend these appointments to understand their condition better and receive a proper diagnosis; (3) concerns and uncertainty, where at the beginning of their healthcare journey, patients may have more questions and concerns about their health, thus may feel a greater need for reassurance, information and guidance, which can drive them to attend the initial appointment; (4) referral or recommendation, where if a healthcare professional or another trusted referral source recommends a specific clinic or specialist, patients may have a higher level of trust and confidence in attending the initial appointment; (5) curiosity and anticipation, where for some individuals, attending their first appointment represents a new experience, and therefore they may be curious about the healthcare process, eager to meet the healthcare provider and hopeful for improvement in their health condition and (6) lastly, access and convenience, where if the initial appointment is scheduled promptly, is conveniently located and accommodates the patient’s availability, they are more likely to attend.

Findings for the diagnostic clinic’s follow-up appointments where patients are seen after the initial appointment for further assessment and management showed that only 47.62% of patients attended their follow-up appointments.

**FIGURE 1:**
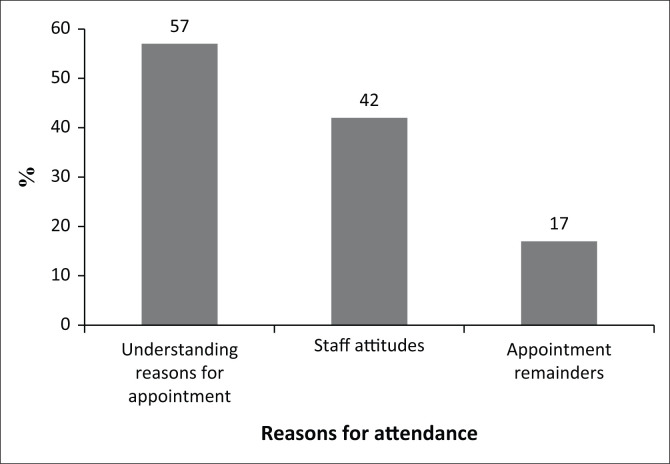
Reasons for attendance.

As far as the reasons for attendance were concerned, Figure 1 displays three key reasons that emerged from the data: understanding reasons for appointment, staff attitudes and appointment reminders.

The first and second most prominent factors noted to contribute to attendance were personal factors such as understanding appointments (57%) and staff attitudes (42%). McLean et al. (2014) conceptualised a framework hypothesising that norms, attitudes and understanding relating to particular conditions or symptoms might shape attendance behaviours, which supports the current findings. The site’s audiology department’s protocol states that patients must be provided with the reason for and the importance of appointments. Understanding the reason for an appointment is dependent on clear informational counselling during audiology consultations (Meibos, 2018).

Staff adherence to the principles of *Batho Pele* was highlighted by patients who reported that a positive attitude of the attending audiologist was a key reason for their attendance. Research has found that establishing rapport with patients is one of the key reasons that patients attend their appointments (Ayat & Ahmed, 2016; Brewster et al., 2020).

Other reasons facilitating return for follow-up appointments in this study included being reminded about the appointment (17%), having access to transport (3%) and living close by to the healthcare facility (3%). A few systematic reviews conducted on the effects of patient reminders found that electronic or telephonic reminders significantly reduced missed appointment rates and improved attendance rates (Henderson, 2008; Opon et al., 2020). Evidence also indicates that reduced barriers to access, such as shorter wait times or flexible appointment scheduling options, can positively impact attendance rates although these were not found to be important factors in the current study.

### Non-attendance and reasons for non-attendance

**FIGURE 2:**
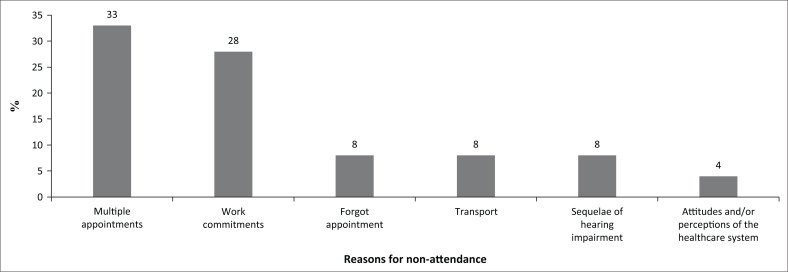
Reasons for non-attendance.

As far as non-attendance was concerned, 52.38% of patients did not attend their follow-up appointments. As stated earlier, poor return rate as opposed to initial appointment rate is a well-documented phenomenon. Six reasons for non-attendance were prominent in the current study: (1) having multiple appointments, (2) work commitments, (3) forgetting the appointment, (4) transport difficulties, (5) attitudes and/or perceptions of the healthcare system and (6) sequelae of hearing impairment as depicted in Figure 2.

The fact that 9 out of 11 patients in this group had chronic medical diagnosis could explain having multiple appointments on the same day as a reason for missing appointments, as reported by 33% of the sample. Evidence suggests that individuals with complex medical conditions that require multiple appointments with different healthcare providers or specialists may struggle to balance numerous appointments, with this becoming overwhelming and leading to missed or rescheduled visits (Wolff et al., 2019). The logistical challenge of coordinating multiple schedules and managing conflicting appointment times can contribute to non-attendance (Marbouh, 2020).

The second most prevalent reason for non-attendance was related to patient employment and caring commitments. Twenty-eight percent of patients reported that commitments such as other appointments, family and work-related matters hindered them from attending their appointments. This has previously been reported by McLean et al. (2014) who described a higher non-attendance at particular times or on particular days that is centred around the incompatibility of the appointment with work and family-related commitments. Work-related responsibilities and commitments can create conflicts with scheduled medical appointments (Mache et al., 2015). Individuals may find it difficult to take time off from work or may fear potential negative consequences, such as salary deductions or job insecurity, which are particularly important factors to consider within the South African context where economic inequalities and unemployment rates are so high (Mache et al., 2015). Balancing work obligations and healthcare needs can be challenging, resulting in missed appointments (Marbouh, 2020).

Forgetting the appointment (8%) and transport difficulties (8%) were the next most prevalent reasons for non-attendance in the current study. Evidence suggests that forgetfulness is a common reason for non-attendance. People lead busy lives with numerous responsibilities, and therefore, it is easy to forget about a scheduled medical appointment, particularly if the appointment is scheduled far in advance or if individuals do not receive reminders or notifications (McLean et al., 2014).

Similarly, limited access to reliable transportation or difficulties in arranging or paying for transportation can be significant barriers to attending medical appointments. Lack of personal transportation, reliance on public transportation with inconvenient schedules or long travel distances or difficulties in finding someone to accompany and assist with transportation can prevent individuals from attending appointments. According to Butler et al. (2001), non-attendance could be because of transport, distance from the hospital and financial factors. Current findings align with this previous evidence as transport and costs pertaining to it were found to be the third highest contributor to non-attendance.

The National Department of Health has proposed a 5 km cluster ruling, which stipulates regulations around where patients can attend public healthcare facilities based on their living proximity to it (Mokgalaka, 2014). Thus, only patients living within the allowed proximity of the research site were participants in this study. While the aim of this regulation was to improve access, there are still associated costs for patients related to public transport. Mokgalaka (2014) found that individuals would rather use public transport, as a 5 km travel distance standard equates to a normal walking time of a maximum of 1 h (Mokgalaka, 2014). This travel time is especially challenging for vulnerable populations who are unable to access the facility on foot, that is, the elderly, the wheelchair-bound and the visually impaired (Green et al., 2015; Mokgalaka, 2014).

Furthermore, 4% of patients presented with findings that highlighted that their attitudes and/or perceptions of the healthcare system in general may have prevented them from attending their audiology appointments. This included factors such as being dissatisfied with the long registration queues, having faced clerical issues such as incorrect bookings and identifying themselves as not requiring audiology services anymore because of a noted improvement in their ear-related diagnoses. In a country such as South Africa, where a vast majority of the population are from the lower socioeconomic stratum, additional factors may influence access and attendance to audiology services. These factors include a lack of coordinated patient care, lack of centralised bookings, excessive waiting times within clinics and poor education for ease of understanding (Khoza-Shangase, 2021).

Generally, literature indicates that attitudes and perceptions of the healthcare system can significantly impact non-attendance at medical appointments in various ways, including: (1) trust and satisfaction – if individuals have negative experiences or perceive a lack of trustworthiness in the healthcare system or specific healthcare providers, they may be less inclined to attend appointments; evidence from studies by Davey et al. (2013), Graham et al. (2015) and Oguro et al. (2021) also suggests that past negative encounters, such as feeling dismissed, rushed or not being heard, can lead to a loss of confidence in the healthcare system and result in non-attendance; (2) communication and information gaps, where effective communication between healthcare providers and patients is not regarded as crucial, thus impacting engagement and attendance; (3) perceived quality of care – if individuals have doubts about the quality or effectiveness of the care they will receive, they may be less motivated to attend appointments; negative perceptions about the healthcare system, such as long waiting times, a perceived lack of expertise or resources or concerns about medical errors, can contribute to non-attendance; (4) cultural and language barriers, where cultural beliefs, language barriers and a lack of cultural sensitivity in healthcare settings influence perceptions of the healthcare system, especially in instances where health-seeking behaviour may be influenced by these cultural beliefs; if individuals feel that their cultural or linguistic needs are not adequately addressed or respected, they may be less likely to attend appointments and (5) accessibility and convenience, where perceptions of the healthcare system’s accessibility and convenience can impact attendance. If individuals perceive the system as difficult to navigate, with long waiting times, limited appointment availability or inconvenient clinic locations, they may be more prone to non-attendance.

According to the WHO (2015), hearing loss causes adults to be excluded from communication causing feelings of loneliness, isolation and frustration. Lin et al. (2013) found that cognitive decline in older adults is associated with hearing loss, which may affect cognitive abilities such as processing speed, working memory and executive functioning, all of which are essential for auditory communication. This sequela of hearing impairment was reported to be the reason for non-attendance for 8% of patients in the current study. These patients specifically scaled it as having a moderate-to-severe impact on their activities of daily living. Cognitive decline can impact healthcare attendance because forgetfulness associated with conditions such as dementia, impaired organisational skills, communication and comprehension challenges, lack of insight into the hearing condition, as well as caregiver burden and coordination, all play a significant role in the elderly patient’s ability to keep their appointment (Powell et al., 2022).

## Conclusion

Current findings have provided factors influencing attendance and non-attendance at an adult audiology clinic at a tertiary hospital in Johannesburg, South Africa. These findings have implications for the service users who are the patients and the service providers who are the healthcare practitioners and support staff. This study reinforces previous research findings that poor attendance can be attributed to difficulties with transportation, employment and personal commitments, distance from the healthcare site and health status, while highlighting that health literacy and *Batho Pele* (people first) ethos by staff positively influence attendance. Because of the negative impacts of non-attendance on early diagnosis and intervention for adults with hearing impairments, the areas needing consolidation and reinforcement in terms of enhancing ear and hearing care (good informational counselling, positive attitudes of staff, appointment reminders, etc.) are also highlighted. These findings, although from one tertiary-level hospital in Gauteng, South Africa, raise important implications for practice. A limitation noted is that the small sample size and specific context limits the generalisability of findings. Therefore, future investigations in other healthcare settings are recommended using more open-ended questions to increase the depth of understanding of issues faced by patients.

## References

[CIT0001] Abuosi, A.A., & Atinga, R.A. (2013). Service quality in healthcare institutions: Establishing the gaps for policy action. *International Journal of Health Care Quality Assurance*, 26(5), 481–492. 10.1108/IJHCQA-12-2011-0077 23905307

[CIT0002] Asvat, H. (2011). *Adherence to attending appointments at Chris Hani Baragwanath Hospital outpatient physiotherapy department* (p. 113). Master’s in, Department of Physiotherapy, University of the Witwatersrand.

[CIT0003] Ayat, A.A.A., & Ahmed, E.G. (2016). Establishing rapport: Physicians’ practice and attendees’ satisfaction at a Primary Health Care Center, Dammam, Saudi Arabia, 2013. *Journal of Family and Community Medicine*, 23(1), 12–17. 10.4103/2230-8229.172224 26929724 PMC4745196

[CIT0004] Boddy, C.R. (2016). Sample size for qualitative research. *Qualitative Market Research*, 19(4), 426–432. 10.1108/QMR-06-2016-0053

[CIT0005] Braun, V., & Clarke, V. (2006). Using thematic analysis in psychology. *Qualitative Research in Psychology*, 3(2), 77–101. 10.1191/1478088706qp063oa

[CIT0006] Brewster, S., Bartholomew, J., Holt, R.G., & Price, H. (2020). Non-attendance at diabetes outpatient appointments: A systematic review. *Diabetic Medicine*, 37(9), 1427–1442. 10.1111/dme.14241 31968127

[CIT0007] Butler, C.W., Snyder, M., Wood, D.E., Curtis, J.R., Albert, R.K., & Benditt, J.O. (2001). Underestimation of mortality following lung volume reduction surgery resulting from incomplete follow-up. *Chest*, 119(4), 1056–1060. 10.1378/chest.119.4.1056 11296169

[CIT0008] Chadha, S., Kamenov, K., & Cieza, A. (2021). The world report on hearing, 2021. *Bulletin of the World Health Organization*, 99(4), 242–242A. 10.2471/BLT.21.285643 33953438 PMC8085630

[CIT0009] Cooksey, R.W. (2020). *Descriptive statistics for summarising data BT – Illustrating statistical procedures: Finding meaning in quantitative data*. Springer Nature

[CIT0010] Davey, A., Asprey, A., Carter, M., & Campbell, J.L. (2013). Trust, negotiation, and communication: Young adults’ experiences of primary care services. *BMC Family Practice*, 14(1), 1–10. 10.1186/1471-2296-14-202 24373254 PMC3880848

[CIT0011] Department of Health. (2018). *Overview*. Retrieved from https://nationalgovernment.co.za/units/view/16/department-of-health-doh

[CIT0012] Dramowski, A., Cotton, M.F., & Whitelaw, A. (2017). A framework for preventing health care-associated infection in neonates and children in South Africa. *South African Medical Journal* 107(3), 192–195. 10.7196/SAMJ.2017.v107i3.12035 28281421

[CIT0013] Education and Training. (2017). *Statistics on post school education and training in South Africa: 2017*. Retrieved from https://www.dhet.gov.za/SiteAssets/Statistics%20on%20Post-School%20Education%20and%20Training%20in%20South%20Africa%20%202017.pdf

[CIT0014] Ethnologue. (2022, July 06). *Ethnologue: Languages of the world*. Retrieved from https://www.ethnologue.com/country/ZA/languages

[CIT0015] Etikan, I., Musa, S.A., & Alkassim, R.S. (2016). Comparison of convenience sampling and purposive sampling. *American Journal of Theoretical and Applied Statistics*, 5(1), 1–4. 10.11648/j.ajtas.20160501.11

[CIT0016] Frost, B.G., Tirupati, S., Johnston, S., Turrell, M., Lewin, T.J., Sly, K.A., & Conrad, A.M. (2017). An Integrated Recovery-oriented Model (IRM) for mental health services: Evolution and challenges. *BMC Psychiatry*, 17(1), 1–17. 10.1186/s12888-016-1164-3 28095811 PMC5240195

[CIT0017] Geiger, S.L. (2015). *Nonattendance rates and barriers to health care in outpatient clinic settings*. Doctoral dissertation. Walden University.

[CIT0018] Graham, J.L., Shahani, L., Grimes, R.M., Hartman, C., & Giordano, T.P. (2015). The influence of trust in physicians and trust in the healthcare system on linkage, retention, and adherence to HIV care. *AIDS Patient care and STDs*, 29(12), 661–667. 10.1089/apc.2015.0156 26669793 PMC4684652

[CIT0019] Green, S., Mophosho, M., & Khoza-Shangase, K. (2015). Commuting and communication: An investigation of taxi drivers’ experiences, attitudes and beliefs about passengers with communication disorders. *African Journal of Disability*, 4(1), a91. 10.4102/ajod.v4i1.91 PMC5433489 28730016

[CIT0020] Guest, G., Bunce, A., & Johnson, L. (2006). How many interviews are enough? An experiment with data saturation and variability. *Field Methods*, 18(1), 59–82. 10.1177/1525822X05279903

[CIT0021] Henderson, R. (2008). Encouraging attendance at outpatient appointments: Can we do more? *Scottish Medical Journal*, 53(1), 9–12. 10.1258/rsmsmj.53.1.9 18422203

[CIT0022] Hugill, K. (2021). *Communication processes between caregivers and audiologists in paediatric cochlear implant appointments in Johannesburg South Africa*. Doctoral dissertation, University of the Witwatersrand.

[CIT0023] Kanji, A., & Khoza-Shangase, K. (2018). In pursuit of successful hearing screening: An exploration of factors associated with follow-up return rate in a risk-based newborn hearing screening programme. *Iranian Journal of Pediatrics* 28(4), a56047. 10.5812/ijp.56047

[CIT0024] Kanji, A., & Krabbenhoft, K. (2018). ‘Audiological follow-up in a risk-based newborn hearing screening programme: An exploratory study of the influencing factors’, *South African Journal of Communication Disorders*, 65(1), a587. 10.4102/sajcd.v65i1.587 PMC6244148 30456962

[CIT0025] Kaur, P., Stoltzfus, J., & Yellapu, V. (2018). Descriptive statistics. *International Journal of Academic Medicine*, 4(1), 60–63. 10.4103/IJAM.IJAM_7_18

[CIT0026] Kheirkhah, P., Feng, Q., Travis, L.M., Tavakoli-Tabasi, S., & Sharafkhaneh, A. (2016). Prevalence, predictors and economic consequences of no-shows. *BMC Health Services Research*, 16(1), 13. 10.1186/s12913-015-1243-z 26769153 PMC4714455

[CIT0027] Khoza-Shangase, K. (2021). Confronting realities to early hearing detection in South Africa. In K. Khoza-Shangase & A. Kanji (Eds.), *Early detection and intervention in audiology: An African perspective* (pp. 66–68). Wits University Press.

[CIT0028] Khoza-Shangase, K. (2022a). In pursuit of increasing the application of tele-audiology in South Africa: COVID-19 puts on the alert for patient site facilitator training. *South African Journal of Communication Disorders*, 69(2), 1–10. 10.4102/sajcd.v69i2.900 PMC9350208 35924605

[CIT0029] Khoza-Shangase, K. (2022b). Complexities and challenges in preventive audiology within the African context. In K. Khoza-Shangase (Ed.), *Complexities and challenges in preventive audiology: An African perspective* (pp. 1–21). AOSIS.

[CIT0030] Khoza-Shangase, K., & Mophosho, M. (2018). Language and culture in speech-language and hearing professions in South Africa: The dangers of a single story. *South African Journal of Communication Disorders*, 65(1), 1–7. 10.4102/sajcd.v65i1.594 PMC6111603 30035607

[CIT0031] Khoza-Shangase, K., & Mophosho, M. (2021). Language and culture in speech-language and hearing professions in South Africa: Re-imagining practice. *South African Journal of Communication Disorders*, 68(1), 9. 10.4102/sajcd.v68i1.793 PMC8252163 34082547

[CIT0032] Lalkhen, H., & Mash, R. (2015). Multimorbidity in non-communicable diseases in South African primary healthcare. *South African Medical Journal*, 105(2), 134–138. 10.7196/SAMJ.8696 26242533

[CIT0033] Lexico. (2021). *Meaning of “Attendance.”* Retrieved from https://www.lexico.com/definition/attendance

[CIT0034] Lin, F.R., Yaffe, K., Xia, J., Xue, Q.L., Harris, T.B., Purchase-Helzner, E., Satterfield, S., Ayonayon, H.N., Ferrucci, L., & Simonsick, E.M. (2013). Hearing loss and cognitive decline in older adults. *JAMA Internal Medicine*, 173(4), 293–299. 10.1001/jamainternmed.2013.1868 23337978 PMC3869227

[CIT0035] Louw, C., Swanepoel, D.W., Eikelboom, R.H., & Hugo, J. (2018). Prevalence of hearing loss at primary health care clinics in South Africa. *African Health Sciences*, 18(2), 313–320. 10.4314/ahs.v18i2.16 30602958 PMC6306974

[CIT0036] Mache, S., Bernburg, M., Vitzthum, K., Groneberg, D.A., Klapp, B.F., & Danzer, G. (2015). Managing work–family conflict in the medical profession: Working conditions and individual resources as related factors. *BMJ Open*, 5(4), e006871. 10.1136/bmjopen-2014-006871 PMC4420985 25941177

[CIT0037] Malakoane, B., Heunis, J.C., Chikobvu, P., Kigozi, N.G., & Kruger, W.H. (2020). Public health system challenges in the free state, South Africa: A situation appraisal to inform health system strengthening. *BMC Health Services Research*, 20(1), 58. 10.1186/s12913-019-4862-y 31973740 PMC6979387

[CIT0038] Maluleke, N.P. (2022). Economic evaluation of EHDI programmes in South Africa: Putting EHDI on the political advocacy and resource allocation agenda. In K. Khoza-Shangase (Ed.), *Preventive audiology: An African perspective* (pp. 199–212). AOSIS Books. 38446947

[CIT0039] Maphumulo, W.T., & Bhengu, B.R. (2019). Challenges of quality improvement in the healthcare of South Africa post-apartheid: A critical review. *Curationis*, 42(1), a1901. 10.4102/curationis.v42i1.1901 PMC6556866 31170800

[CIT0040] Marbouh, D., Khaleel, I., Al Shanqiti, K., Al Tamimi, M., Simsekler, M.C.E., Ellahham, S., Alibazoglu, D., & Alibazoglu, H. (2020). Evaluating the impact of patient no-shows on service quality. *Risk Management and Healthcare Policy*, 13, 509–517. 10.2147/RMHP.S232114 32581613 PMC7280239

[CIT0041] Meibos, A.R. (2018). *Counseling competencies in audiology: Important knowledge, skills, and attitudes*. Doctoral dissertation, Utah State University.

[CIT0042] McLean, S., Gee, M., Booth, A., Salway, S., Nancarrow, S., Cobb, M., & Bhanbhro, S. (2014). *Patterns and influences on health-care attendance behaviour: A narrative overview of key themes and issues*. NCBI. Retrieved from https://www.ncbi.nlm.nih.gov/books/NBK260108/

[CIT0043] Mokgalaka, H. (2014). Measuring access to primary health care: Use of a GIS-based accessibility analysis. Planning Africa 2014 Conference, July. Retrieved from http://researchspace.csir.co.za/dspace/bitstream/10204/7913/1/Mokgalaka_2014.pdf

[CIT0044] Morse, J.M. (2000). Determining sample size. *Qualitative Health Research*, 10(1), 3–5. 10.1177/104973200129118183

[CIT0045] Mulwafu, W., Kuper, H., & Ensink, R.J.H. (2016). Prevalence and causes of hearing impairment in Africa. *Tropical Medicine and International Health*, 21(2), 158–165. 10.1111/tmi.12640 26584722

[CIT0046] Naidoo, S. (2012). The South African national health insurance: A revolution in health-care delivery! *Journal of Public Health*, 34(1), 149–150. 10.1093/pubmed/fds008 22362968

[CIT0047] National Department of Health. (2020). *Strategic plan 2020/21–2024/25*. Retrieved from https://www.health.gov.za/wp-content/uploads/2020/11/depthealthstrategicplanfinal2020-21to2024-25-1.pdf

[CIT0048] Oguro, N., Suzuki, R., Yajima, N., Sakurai, K., Wakita, T., Hall, M.A., & Kurita, N. (2021). The impact that family members’ health care experiences have on patients’ trust in physicians. *BMC Health Services Research*, 21(1), 1–11. 10.1186/s12913-021-07172-y 34666754 PMC8527743

[CIT0049] Opon, S.O., Tenambergen, W.M., & Njoroge, K.M. (2020). The effect of patient reminders in reducing missed appointment in medical settings: A systematic review. *Pan African Medical Journal One Health*, 2(9), 1–10. 10.11604/pamj-oh.2020.2.9.21839

[CIT0050] Peter, V.Z., Paken, J., & Joseph, L. (2020). An audiological profile of a cohort of school-aged children with HIV and AIDS attending an antiretroviral clinic in South Africa. *South African Journal of Communication Disorders*, 67(1), 1–9. 10.4102/sajcd.v67i1.651 PMC7203215 32370522

[CIT0051] Pillay, M., Tiwari, R., Kathard, H., & Chikte, U. (2020). Sustainable workforce: South African audiologists and speech therapists. *Human Resources for Health*, 18(1), 1–13. 10.1186/s12960-020-00488-6 32611357 PMC7329495

[CIT0052] Powell, D.S., Oh, E.S., Reed, N.S., Lin, F.R., & Deal, J.A. (2022). Hearing loss and cognition: What we know and where we need to go. *Frontiers in Aging Neuroscience*, 821. 10.3389/fnagi.2021.769405 PMC8920093 35295208

[CIT0053] Ramma, L., & Sebothoma, B. (2016). The prevalence of hearing impairment within the Cape Town Metropolitan area. *The South African Journal of Communication Disorders = Die Suid-Afrikaanse Tydskrif Vir Kommunikasieafwykings*, 63(1), a105. 10.4102/sajcd.v63i1.105 PMC5843235 27247255

[CIT0054] Staley, H., Shiraz, A., Shreeve, N., Bryant, A., Martin-Hirsch, P.P., & Gajjar, K. (2021). Interventions targeted at women to encourage the uptake of cervical screening. *Cochrane Database of Systematic Reviews*, 9, CD002834. 10.1002/14651858.CD002834.pub3 34694000 PMC8543674

[CIT0055] Statistics South Africa. (2022). *Census 2022*. Retrieved from https://www.statssa.gov.za/?m=2022

[CIT0056] Stellenberg, E.L. (2015). Accessibility, affordability and use of health services in an urban area in South Africa. *Curationis*, 38(1), a102. 10.4102/curationis.v38i1.102 PMC6091258 26016516

[CIT0057] Thompson, A.E., Anisimowicz, Y., Miedema, B., Hogg, W., Wodchis, W.P., & Aubrey-Bassler, K. (2016). The influence of gender and other patient characteristics on health care-seeking behaviour: A QUALICOPC study. *BMC Family Practice*, 17(1), 1–7. 10.1186/s12875-016-0440-0 27036116 PMC4815064

[CIT0058] Van der Spuy, T., & Pottas, L. (2008). Infant hearing loss in South Africa: Age of intervention and parental needs for support. *International Journal of Audiology*, 47(suppl. 1), S30–S35. 10.1080/14992020802286210 18781511

[CIT0059] Wolff, D.L., Waldorff, F.B., Von Plessen, C., Mogensen, C.B., Sørensen, T.L., Houlind, K.C., Bogh, S.B., & Rubin, K.H. (2019). Rate and predictors for non-attendance of patients undergoing hospital outpatient treatment for chronic diseases: A register-based cohort study. *BMC Health Services Research*, 19(1), 1–11. 10.1186/s12913-019-4208-9 31200720 PMC6570866

[CIT0060] World Health Organization. (2015). *World health statistics 2015*. World Health Organization. Retrieved from https://apps.who.int/iris/handle/10665/170250

[CIT0061] Young, M. (2016). *Private vs. public healthcare in South Africa [Western Michigan University.]*. Honors Thesis: Vol. Paper 2741. Retrieved from http://scholarworks.wmich.edu/honors_theses

